# Cardiac Arrhythmia Classification Using Advanced Deep Learning Techniques on Digitized ECG Datasets

**DOI:** 10.3390/s24082484

**Published:** 2024-04-12

**Authors:** Shoaib Sattar, Rafia Mumtaz, Mamoon Qadir, Sadaf Mumtaz, Muhammad Ajmal Khan, Timo De Waele, Eli De Poorter, Ingrid Moerman, Adnan Shahid

**Affiliations:** 1 School of Electrical Engineering and Computer Science (SEECS), National University of Sciences and Technology (NUST), Islamabad 44000, Pakistan; ssattar.mscs19seecs@seecs.edu.pk (S.S.); ajmal.khan@seecs.edu.pk (M.A.K.); 2 Federal Government Poly Clinic Hospital, Islamabad 44000, Pakistan; drmamoonqadir@yahoo.com; 3 NUST School of Health Sciences (NSHS), National University of Sciences and Technology (NUST), Islamabad 44000, Pakistan; sadaf.mumtaz@nshs.nust.edu.pk; 4 IDLab, Department of Information Technology, Ghent University-IMEC, 9052 Ghent, Belgium; timo.dewaele@ugent.be (T.D.W.); eli.depoorter@ugent.be (E.D.P.); ingrid.moerman@ugent.be (I.M.); adnan.shahid@ugent.be (A.S.)

**Keywords:** ECG classification, deep learning, digitization, arrhythmia, self-supervised learning

## Abstract

ECG classification or heartbeat classification is an extremely valuable tool in cardiology. Deep learning-based techniques for the analysis of ECG signals assist human experts in the timely diagnosis of cardiac diseases and help save precious lives. This research aims at digitizing a dataset of images of ECG records into time series signals and then applying deep learning (DL) techniques on the digitized dataset. State-of-the-art DL techniques are proposed for the classification of the ECG signals into different cardiac classes. Multiple DL models, including a convolutional neural network (CNN), a long short-term memory (LSTM) network, and a self-supervised learning (SSL)-based model using autoencoders are explored and compared in this study. The models are trained on the dataset generated from ECG plots of patients from various healthcare institutes in Pakistan. First, the ECG images are digitized, segmenting the lead II heartbeats, and then the digitized signals are passed to the proposed deep learning models for classification. Among the different DL models used in this study, the proposed CNN model achieves the highest accuracy of ∼92%. The proposed model is highly accurate and provides fast inference for real-time and direct monitoring of ECG signals that are captured from the electrodes (sensors) placed on different parts of the body. Using the digitized form of ECG signals instead of images for the classification of cardiac arrhythmia allows cardiologists to utilize DL models directly on ECG signals from an ECG machine for the real-time and accurate monitoring of ECGs.

## 1. Introduction

Heart diseases, also known as cardiovascular diseases (CVDs), are the most common and leading causes of mortality worldwide. In the year 2019, approximately 18 million deaths were attributed to CVDs, which was 32% or nearly one-third of total deaths globally [1]. Diagnosing heart disease in its early stages can save a large number of precious lives. Advancements in technology, especially artificial intelligence (AI), have revolutionized every domain of life, including healthcare, where it is assisting human experts in diagnosis, treatment, and medical care [2]. In heart disease diagnostics or cardiology, electrocardiograms (ECGs) play a pivotal role in providing necessary information about heart conditions. The manual analysis of ECGs is not only time-consuming but also requires a high level of human expertise that only comes after years of experience. Thus, an accurate mechanism for the automated analysis of ECGs is crucial for improving and expanding quality healthcare services for patients with CVDs.

**Figure 1:**
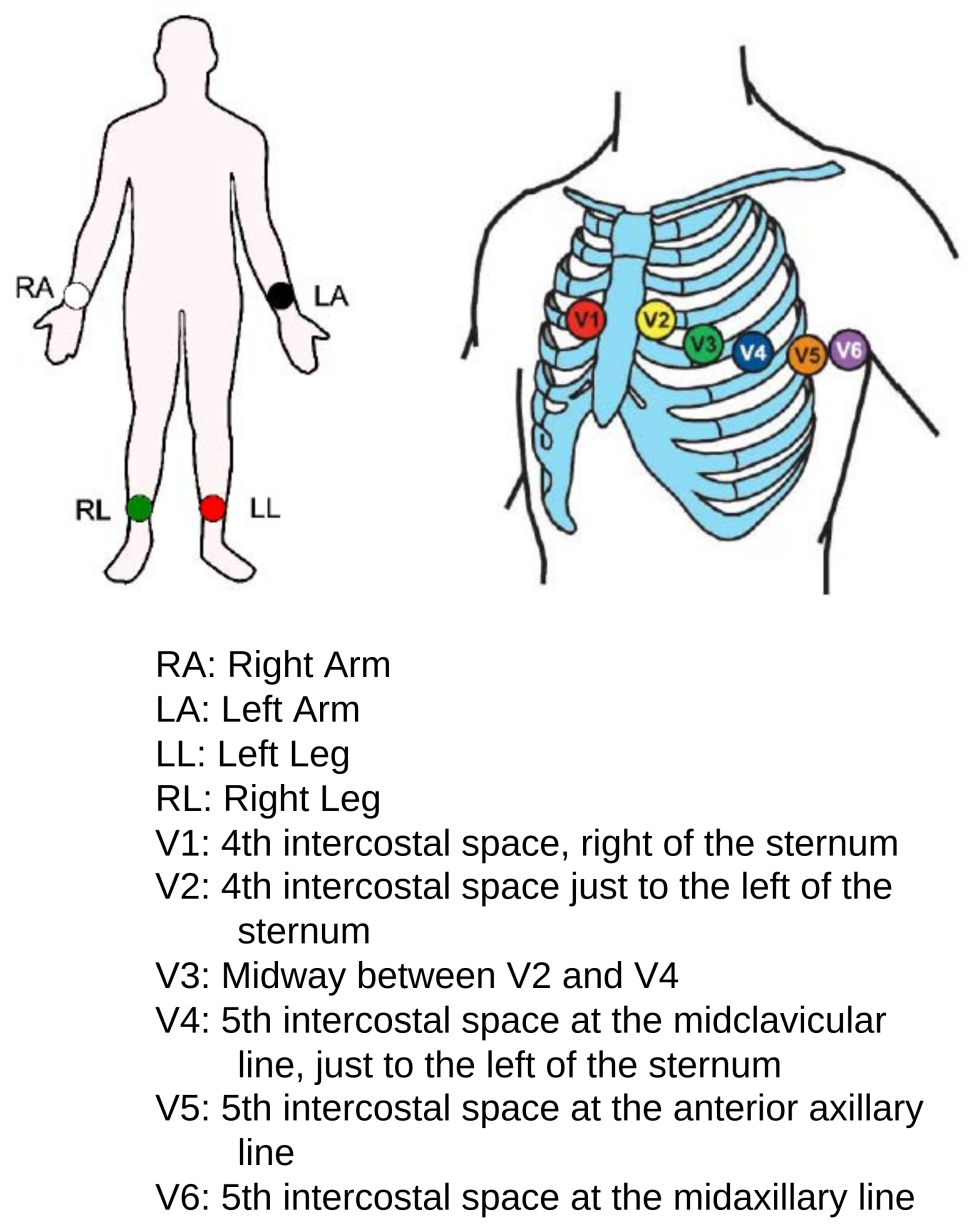
Placement of electrodes on the body for a 12-lead ECG [7].

**Figure 2:**
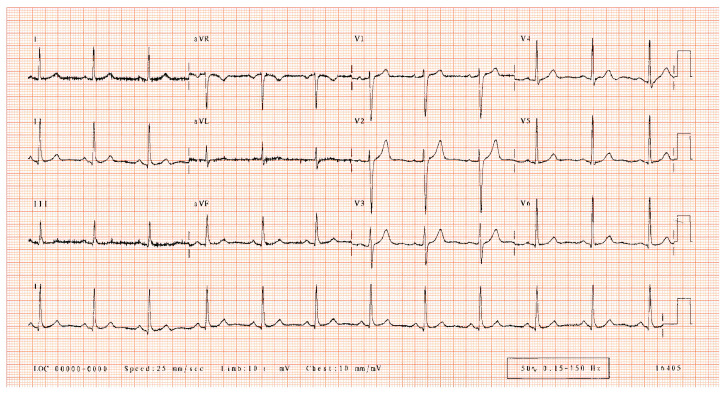
A 12-lead ECG record of a normal person showing heartbeats from a combination of different electrodes [8].

**Figure 3:**
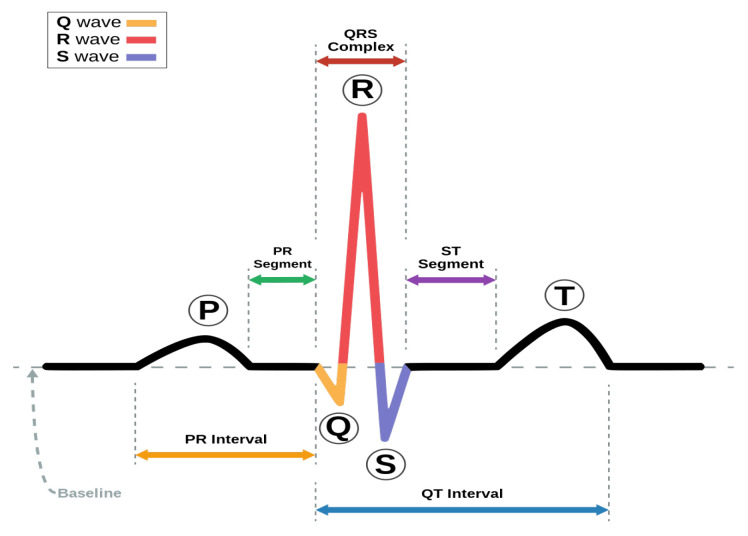
A normal ECG heartbeat showing different waves and segments [9].

An ECG is the main source of information about the heartbeat rhythm that helps medical experts or cardiologists quickly screen patients for heart diseases. An ECG represents the electrical activity of the heart in the form of a waveform graph recorded through electrodes placed on different parts of the body (arms, chest, and legs). A specific combination of these electrodes is known as a lead. The simplest lead is determined by two electrodes, in which one electrode acts as a positive and the other one as a negative, but in general, two or three electrodes in combination form a lead. The most common standard is a 12-lead ECG, which is recorded using 10 electrodes. The placement of various electrodes on the body for a 12-lead ECG is shown in Figure 1, and the output of a 12-lead ECG of a healthy person is shown in Figure 2 on graph paper. A heartbeat, also called a cardiac cycle, has different components in an ECG plot [3], as shown in Figure 3. In a complete heartbeat, the P wave represents the contraction of the heart, or atrial depolarization; the QRS complex represents ventricular depolarization; and the T wave shows the relaxation phase, or ventricular repolarization. The heartbeat’s structures provide valuable information about the heart’s condition and functioning, assisting healthcare providers in diagnosing various cardiac conditions, such as arrhythmias and cardiac diseases [4,5,6].

ECG classification is an important task in cardiology and biomedical research [10]. It involves classifying an ECG or the heartbeats into different heart conditions based on the structure of the heartbeats from one or multiple leads. The most simple classification task performed on an ECG is to classify its output into binary classes, i.e., normal vs. abnormal [11]. However, there are multiple arrhythmia conditions and heart diseases that need to be individually identified for accurate diagnosis [3]. ECG classification plays a significant role in arrhythmia detection, assessing the risk of heart diseases, and analyzing fitness and performance in sports [12]. However, the main focus in this work is on arrhythmia detection and the classification of heartbeats in different cardiac conditions. Many datasets of ECG data have been created for research and development purposes, and these have helped in exploring various machine learning (ML) models for ECG classification.

Machine learning (ML) has shown significant contributions in healthcare and medical diagnostics, such as in cancer detection methods that use image segmentation of CT scans, MRIs, and X-rays [2]. Similarly, in the classification of cardiac abnormalities, ML has shown promising results [3,13,14]. Early applications of machine learning for ECG classification employed various traditional machine learning techniques, including support vector machines (SVM) [15,16] and k-nearest neighbors (kNN) [17]. As the field of AI has developed, more advanced deep learning-based techniques, such as convolutional neural networks (CNNs) [3], long short-term memory (LSTM) networks [18], and transformer neural networks [19,20] have been explored for the analysis of ECG signals. The classification of ECG data has many important applications, including but not limited to diagnosis in the absence of a cardiologist, automatic verification of ECG reports, and helping medics in teaching [21].

In this work, ECG recordings from a dataset collected from various healthcare units in Pakistan are utilized for the classification of different arrhythmias and cardiac diseases. The dataset, known as the CPEIC cardiac dataset [22], contains images of ECG records, which, in this work, are converted to numerical form using an open-source digitization tool [23] by segmenting the Lead II heartbeats. The details of the digitization tool, known as *ecg_digitize*, are discussed in Section 3.2. The digitized signals are then pre-processed and fed to the deep learning model for the classification of four cardiac classes. Deep learning models are capable of learning complex patterns in data. Since ECG signals contain intricate patterns, deep learning is helpful in learning these patterns with state-of-the-art performance. Similarly, deep learning models generalize well on different patients’ data, thus providing accurate predictions on new records. Novel deep learning architectures are developed, which include a proposed CNN, an LSTM, and an SSL-based method using autoencoders. Training the models on digitized signals allows them to be used directly on the ECG signals that are generated from the ECG machine using electrodes/sensors so that direct monitoring of the ECG can be conducted. Thus, the work presented contributes to an important application of deep learning on sensors. The main contributions of this work are:

- The use of a digitized time series data format of the CPEIC cardiac dataset, which hitherto has not been used in its digitized form for ECG classification.
- The fast inference and classification of ECG in real time using only data from heartbeats from lead II.
- The proposed models can be deployed on an ECG machine for the near-real-time monitoring and analysis of the ECG signals.
- The proposed CNN model achieves competitive results on the CPEIC dataset with a high accuracy of ∼92% on four classes.

The paper is organized as follows. Section 2 summarizes the latest techniques in the literature for ECG classification tasks. Section 3 explains the proposed methodology, along with the datasets and pre-processing in detail. The results are discussed in Section 4, and the paper is concluded with future recommendations in Section 5.

## 2. Related Work

Many machine learning and deep learning techniques have been reported in the literature for classifying ECG data or heartbeats into different cardiac arrhythmia classes. The machine learning methods include a decision tree classifier, random forest, and k-nearest neighbors. The deep learning methods can be classified into convolutional neural networks, sequence models, attention mechanisms, zero-shot learning, and self-supervised learning methods.

### 2.1. Machine Learning Approaches

In the traditional machine learning approach, the decision tree classifier is used in many research works. An optimized decision tree classification model was proposed by Kumari et al. [24] to classify six different classes of heartbeats, where one class was normal and the other five classes represented some arrhythmia. After pre-processing the data, the researchers extracted 17 morphological and 7 temporal features to feed to the classification model. An optimized decision tree classifier with an adaptive boosting mechanism was then used for classification. The decision tree was optimized to handle imprecise, uncertain, and incomplete data. The authors reported the best accuracy of 98.77% and a 93.85% F1 score on the MIT-BIH arrhythmia dataset with six selected classes.

Bhattacharyya et al. [25] proposed an ensemble of a support vector machine (SVM) and random forest for heartbeat classification, which were combined using a weighted majority algorithm. Five classes from the MIT-BIH arrhythmia dataset were used in this study, including one normal class and four abnormal classes. A time series feature extraction library (TSFEL) [26] was used to extract 61 features from the data. Feature selection techniques were employed as well, such as feature scaling, the removal of low-variance and highly correlated features, and recursive feature elimination. The synthetic minority oversampling technique (SMOTE) [27] was used for oversampling to handle class imbalances. The proposed model demonstrated an accuracy of 98.21% and an F1 score of 96.4% on the five classes from the MIT-BIH arrhythmia dataset, which were normal (N), ventricular ectopic beat (VEB), supra-ventricular ectopic beat (SVEB), unclassifiable beat (Q), and fusion of a ventricular and normal beat (F).

Zou et al. [28] proposed a novel method for feature extraction for improved heartbeat classification. A feature called *segment label* was learned from a convolutional neural network, which was then combined with other traditional features and input to a random forest classifier. The model was trained on the MIT-BIH arrhythmia dataset for classifying three classes of heartbeats; N, VEB, and SVEB. For these three classes, this approach achieved an accuracy of 96% and an F1 score of 88.3%. This study showed the contribution of *segment label* in accurately classifying heartbeats, particularly in cases where rhythm information serves as essential contextual information.

Ahmed et al. [29] proposed an ensemble of different machine learning models, including a kNN, decision tree, artificial neural network (ANN), support vector machine (SVM), and LSTM. The hard voting method was used for ensembling. Class imbalance was also handled using class weights, which assigned greater emphasis to the minority class by assigning higher weights while allocating lower weights to the majority class. The performance of the ensemble was measured on two datasets: the Physikalisch Technische Bundesanstalt (PTB) Diagnostic ECG dataset and the MIT-BIH arrhythmia dataset, with two and five classes, respectively. The proposed approach demonstrated accuracies of 98.06% and 97.664% on the MIT-BIH and PTB datasets, respectively.

A summary of different machine learning approaches is shown in Table 1.

### 2.2. Deep Learning Approaches

Deep learning approaches perform an end-to-end classification of ECGs. This is in contrast to traditional machine learning approaches, where feature extraction must be carried out before training the model [30]. Cutting-edge deep learning techniques for ECG classification are discussed below to gain insight into the ongoing research and development in the field.

CNNs are among the most important and widely used deep learning models. CNN-based models contain many layers that transform their inputs into valuable representations with convolution filters. They are particularly effective in capturing local patterns and spatial dependencies within the data [31]. CNNs have the capacity for hierarchical representation learning, capturing complex patterns from the input data [32]. CNNs have been recently used in the classification of ECG signals [31], giving improved performance.

Recently, Qureshi et al. [3] proposed a deep learning approach using CNNs to classify multiple classes of heartbeats and achieved robustness by incorporating dropout and early stopping. The issue of class imbalance was addressed through the utilization of SMOTE. The model outperformed baseline models, achieving approximately 96% accuracy and very fast inference of less than one second. The proposed method demonstrated generality by classifying ten classes accurately.

A two-phase approach was proposed by Bruoth et al. [21] for ECG classification on the PhysioNet 2021 challenge dataset. In phase 1, a base model was trained using data from different sources. The 1D version of ResNet-50 was used as the base model. In phase 2, the base model was fine-tuned on the challenge metrics and conditions. The authors also modified the labels in this study into three labels and used a flow-mixup layer to add random convex combinations to the input data. They used different lead configurations, including 2 leads, 3 leads, 4 leads, 6 leads, and 12 leads, and applied various techniques, such as data augmentation and wavelet transformation, to pre-process the ECG signals. In the training phase, 10-fold cross-validation was used, and the best model was selected based on the micro-F2 score obtained on the validation set. The final model was an ensemble of 10 neural networks. During the training, the authors employed the AdamW optimizer with a weight decay rate of 0.0005, utilizing a batch size of 128. The proposed method was computationally efficient and achieved 52% on the challenge metric, which is a generalized form of accuracy. The authors claimed that there is no advantage of using data sampled at 500 Hz compared to 100 Hz and no benefit of extending the input size beyond 3 s.

Ahmed et al. [33] proposed a 1D CNN for the classification of cardiac arrhythmias. The proposed method incorporated noise in the MIT-BIH arrhythmia dataset for training to make the model perform better on noise-attenuated signals. Lead II heartbeats were extracted to train the model to classify them in 4 arrhythmic classes. The model achieved outstanding results, with the authors reporting an accuracy of 99% on the test set. The recall and specificity were 94% and 99%, respectively.

An ensemble approach was presented by Mahmud et al. [34] that utilized a 1D CNN on ECG signals and a 2D CNN on ECG images using transfer learning. The predictions were combined using an ensemble technique, providing commendable results. On the ECG signals, the proposed approach achieved 94% accuracy, and on the ECG images, it achieved 93% accuracy on 5 classes. Data augmentation was employed on the images to improve the results.

A famous sequence model is long short-term memory (LSTM), which is a type of recurrent neural network (RNN) designed to handle patterns in sequential and time series data. LSTM networks consist of memory cells that can store and retrieve information over extended sequences, enabling them to retain important information for later use [35]. Karri et al. [36] proposed an LSTM network for ECG classification on the MIT-BIH arrhythmia database. The features were extracted and hybridized to feed to the LSTM network for classification. A method of detecting the QRS complex was devised using discrete wavelet transform and delta sigma modulation. The proposed method showed an accuracy of 99.64% and an F1 score of 98.18% on five classes from the MIT-BIH dataset.

An LSTM-based ECG classification model was used on compressed features of ECG signals by Yildirim et al. [37]. The ECG signals were passed through a convolutional autoencoder to obtain a compressed representation of the data in order to reduce the computation time during classification. The features extracted using the encoder part of the autoencoder were then fed to the LSTM network for classification. The LSTM network trained on the encoded features demonstrated high accuracy on five classes of the MIT-BIH dataset. The accuracy and F1 score both were ∼99%.

Transformer networks extensively use the attention mechanism as a fundamental component of their architecture. The attention mechanism in the transformer is called self-attention or scaled dot-product attention [38]. A transformer neural network was developed for the classification of 12-lead ECG signals in [20]. The model classified the ECG signals from the PhysioNet-2020 challenge into 27 classes using 12-lead data. Hand-crafted features using random forests were combined with a transformer network’s discriminative features. The model was trained using 10-fold nested cross-validation. The model performed best on the challenge metric and achieved a score of 0.53 on the test data. The challenge metric is a generalized form of accuracy. During pre-processing, ECG records were upsampled or downsampled to obtain a fixed sampling rate of 500 Hz for consistency. A finite impulse response bandpass filter with a bandwidth of 3–45 Hz was also applied to remove any unwanted noise or artifacts from the ECG signal. Normalization was carried out to ensure each record had zero mean and unit variance.

Yamaç et al. [39] developed a personalized arrhythmia detection system using one-shot learning for wearable devices. The proposed method used null space analysis on the normal heartbeats, which made it computationally efficient. It also used domain adaptation using sparse representation to map existing users’ information onto new users, enabling the classification of normal vs. abnormal heartbeats without the need for abnormal heartbeats from new users. Combining the null space analysis and domain adaptation, the authors proposed an ensemble classifier that yielded 98.2% accuracy and an F1 score of 92.8% on the MIT-BIH dataset in normal vs. abnormal scenarios.

Phan et al. [14] proposed an SSL-based multi-modality method for ECG classification that used self-distillation without labels. There were two tasks in this method: a pre-stream task and a downstream task. In the pre-stream task, a 1D CNN for time series data and a 2D CNN for time–frequency spectral signals were used and trained on unlabeled data. In the downstream task, the 1D and 2D CNNs were fine-tuned on labeled data. The features of each CNN were fused using a proposed gated fusion mechanism. The proposed method demonstrated an accuracy of 48.9% and an F1 score of 62.1 on the test set of the PhysioNet 2020 challenge dataset. A summary of all the deep learning-based models that are reviewed in this study is presented in Table 2.

Our main focus in this study is to train machine learning and deep learning models on a digitized form of the CPEIC cardiac dataset. All of the existing approaches implemented on this dataset use models directly on images, which hinders the use of techniques that are applied to time series data, such as MIT-BIH. Secondly, there are some important cardiac arrhythmia classes in the CPEIC cardiac dataset, but the dataset is available only in image format, so converting it to a time series format gives us a numeric dataset with those cardiac disease classes. In the following section, the proposed approach is discussed in detail for classifying cardiac diseases on the digitized form of the CPEIC cardiac dataset using both machine learning and deep learning techniques.

## 3. Proposed Methodology

**Figure 4:**
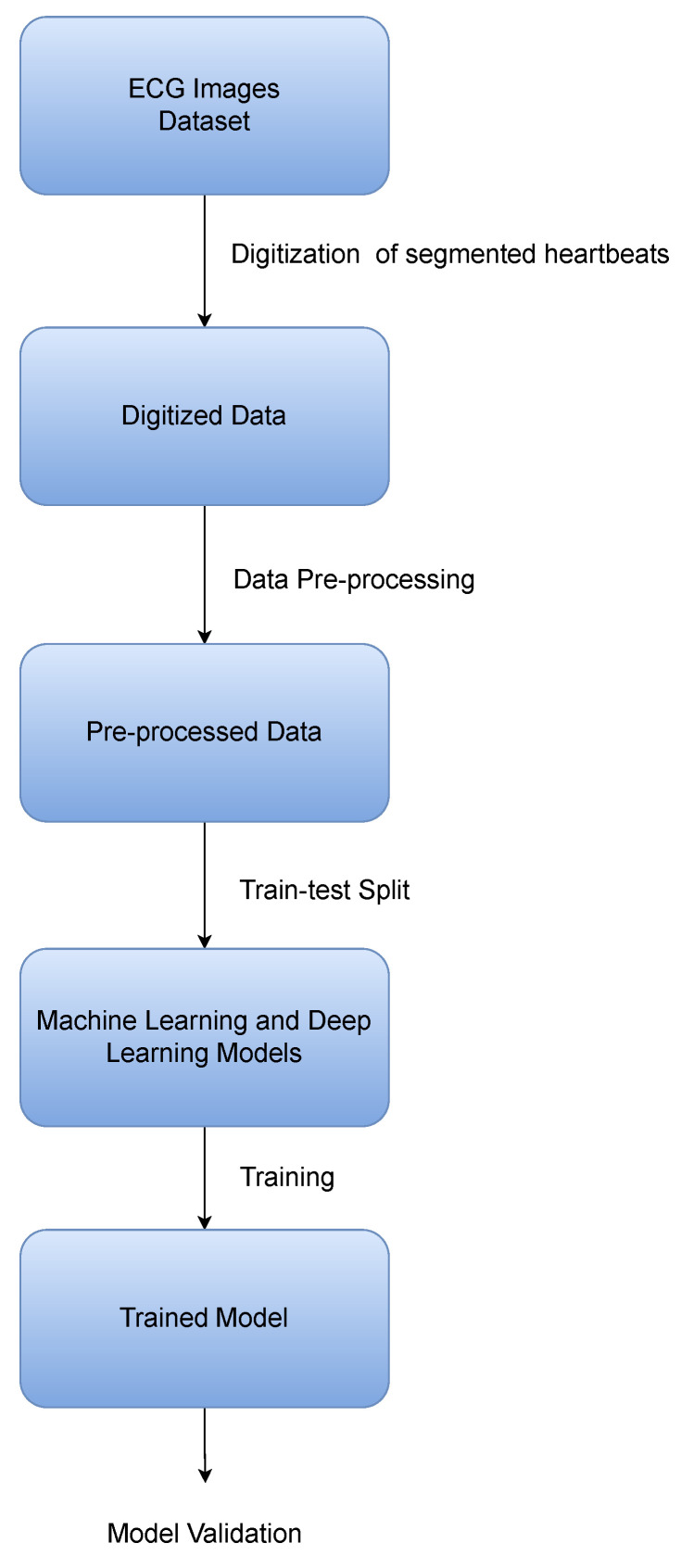
Overall pipeline of the ECG classification workflow.

The accurate and timely classification of abnormalities in the heart and arrhythmias is a crucial first step in cardiac healthcare. To accurately classify these abnormalities using machine learning is a challenging task and becomes even more challenging when the dataset size is small [44]. In this work, multiple architectures using novel deep learning techniques are proposed for the classification of cardiac diseases on the basis of heartbeat data extracted from ECG records. The overall pipeline of the proposed methodology is shown in Figure 4. This section discusses in detail the major building blocks of the pipeline, which include:

- The datasets used for ECG classification;
- The digitization tool used to digitize the ECG images;
- The pre-processing method used to transform the data into a standardized form;
- The architecture and the models used to obtain accurate results on the CPEIC cardiac dataset;
- Lastly, the classes that the models are trained to classify.

### 3.1. Datasets

#### 3.1.1. MIT-BIH Arrhythmia Database

The MIT-BIH arrhythmia database [45] is a benchmark dataset in ECG classification that was released in 1980 by a collaboration of the laboratories at the Massachusetts Institute of Technology (MIT) and the Beth Israel Hospital (BIH) in Boston. The dataset was collected from 47 subjects. The dataset comprises 48 ECG recordings, each spanning half an hour. The recordings were obtained at a sampling rate of 360 Hz, capturing data with 11-bit resolution within a range of 10 mV. There are 19 beat annotations that represent different classes of heartbeats. The labels and descriptions of these heartbeat types are shown in Table 3.

In this work, the MIT-BIH dataset is used only to augment the CPEIC cardiac dataset in the SSL-based approach. Lead II heartbeats from the MIT-BIH dataset are used without labels to train the pre-stream task in our SSL-based approach. The details are discussed in Section 3.5.3.

#### 3.1.2. ECG Image Dataset of Cardiac Patients

This dataset [22] contains ECG images collected from various healthcare institutes in Pakistan under the supervision of the Chaudhry Pervaiz Elahi Institute of Cardiology (CPEIC), Multan. The ECG images are 12-lead ECG records, which are recorded at a sampling rate of 500 Hz. Each ECG record belongs to a different patient, which helps to increase the deep learning models’ generalization. The dataset is divided into four different classes, of which one is the normal class and the other three are related to cardiac diseases. The dataset is open-source and publicly available (https://data.mendeley.com/datasets/gwbz3fsgp8/2, accessed on 1 August 2023). A summary of the dataset is listed in Table 4.

In this work, instead of directly using images for classification, ECG images were first converted to numerical time-series format using an open-source digitization tool. The reason for using digitized data instead of images and the details of the digitization process are discussed in the following section.

### 3.2. Digitization

ECG images can be used as they are for training a classification model [40,41,42], but these models cannot be used directly on ECG machines for real-time monitoring and prediction. The temporal correlation of ECG signals is also lost in image-based models, and these models require a larger number of parameters to train, thus increasing their computational complexity. Therefore, the ECG images were converted to numerical/time series signals by leveraging open-source ECG digitization tools. Table 5 compares the benefits and limitations of using ECG images and time series signals for ECG classification.

For the digitization of the ECG images from the CPEIC cardiac dataset, an open-source tool, *ecg_digitize* (https://github.com/Tereshchenkolab/ecg-digitize, accessed on 1 August 2023), was used [23]. On the backend, *ecg_digitize* employs image processing techniques to extract signals from images. It first detects and extracts the grid from the ECG image, and then the ECG signal is detected and extracted. In this tool, one can select the region that is required to be digitized. For this study, only heartbeats from lead II were selected by selecting one heartbeat from each image, as the heartbeats are consistent. The digitized signals were saved as CSV files. The reason for using this tool was because of its high correlation of 0.977 and accurate mapping of signals from images. Since the region is selected manually, it performs better than other open-source tools that produce undesirable noise and wrong signals while working end-to-end [46]. For every image, one CSV file was saved for the digitized heartbeat of lead II, and this yielded 928 CSV files at the end of the digitization process.

**Figure 5:**
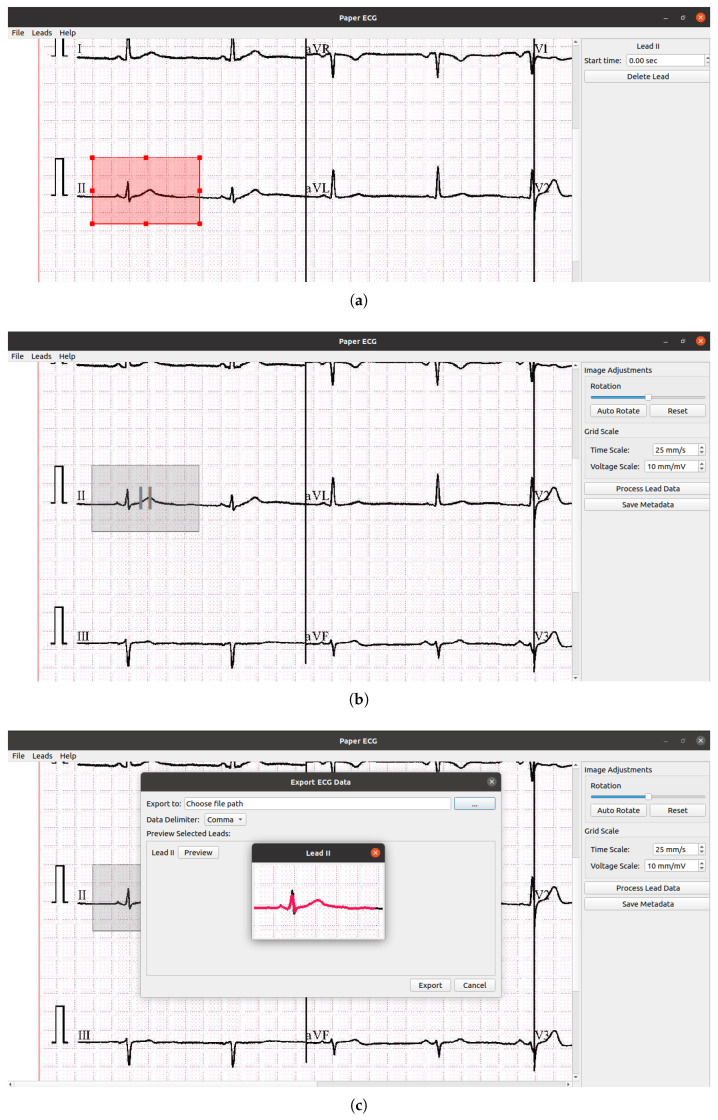
ECG digitization using *ecg_digitize*. (**a**) Select the lead II heartbeat for digitization. (**b**) Adjust the image if tilted. Set the time and voltage, then process. (**c**) Export the digitized ECG heartbeat in a CSV file.

The user interface of ecg_digitze is shown in Figure 5. The digitizing of ECG images using the ecg_digitize tool involved the following steps:

1. In the first step, the image was opened in the digitization app, and lead II was selected from the option “leads” from the top left of the panel. Next, the bounding box was placed on the lead II heartbeat. A complete heartbeat spanned 0.25 s before the R-peak to 0.4 s after the R-peak [3]. The size of the bounding box could be adjusted based on the start and end of the heartbeat.
2. The second step comprised the selection of the time scale and voltage scale of the ECG plot. The image could be adjusted if the ECG plot was rotated or tilted.
3. The processed data were shown in a small window, and the digitized data were then saved in the CSV file format.

**Figure 6:**
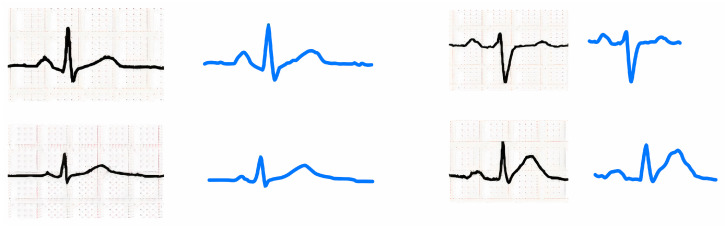
Original images (black lines) and the corresponding digitized signals (blue lines).

Figure 6 shows a few examples of original ECG signals and recreated signals from digitized heartbeat data from lead II. It is obvious that digitized data can reproduce original heartbeats with high fidelity.

### 3.3. Pre-Processing

Pre-processing is a crucial step in the ECG classification workflow. The signals may be of different sampling rates, can have different amplitudes, and may contain noise. The digitization step produced 928 records in the CSV file format, and these digitized ECG signals were pre-processed to make the data consistent, concise, and suitable for training the proposed models. The following pre-processing steps were performed on the CPEIC cardiac dataset.

#### 3.3.1. Interpolation

The digitized values were of different lengths because of the different shapes of heartbeats and minor differences in the sizes of the selected regions. To make all the digitized signals of the same length feature vector, linear interpolation was applied.

(1) $$y=y_{1}+\frac{\left(x-x_{1}\right)\cdot \left(y_{2}-y_{1}\right)}{x_{2}-x_{1}}$$

Following the same feature length of the segmented heartbeats as in [3], the digitized heartbeat signals were interpolated to make their length 234. This made each digitized heartbeat of the same length feature vector.

#### 3.3.2. Noise Removal

**Figure 7:**
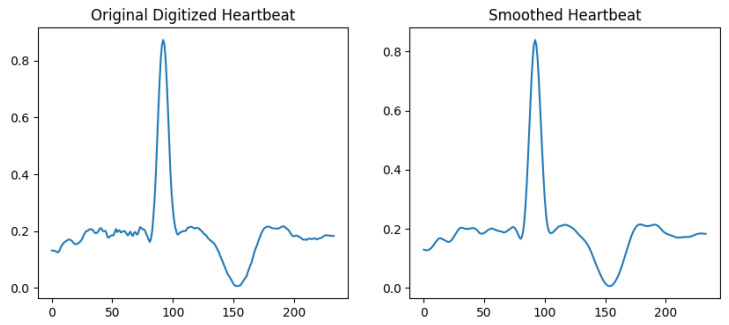
ECG heartbeat before and after smoothing.

In some of the digitized signals, there were noise or jittering at some points. This could be due to blurry images or some noisy pixels. A Savitzky–Golay smoothing filter [47] was applied to make the signals smoother. There are many other methods for data smoothing, such as Gaussian smoothing and exponential smoothing, but the Savitzky–Golay filter performs local polynomial regression on the data, allowing it to capture more complex trends and patterns. It also preserves the shape of the signal better than other smoothing operations, especially when the data contain sharp edges or sudden changes. Figure 7 shows the comparison of a digitized heartbeat before and after applying the smoothing filter.

#### 3.3.3. Normalization/Standardization

The amplitude of the digitized ECG heartbeats can be on a random scale. Therefore, it was necessary to scale the heartbeats on a standard scale so that the model learns the features correctly. For this purpose, we could normalize the beats between 0 and 1 or apply standard scaling. The formula for 0–1 normalization is given below:

(2) $$\begin{array}{c} x_{\mathrm{normalized}}=\frac{x-x_{\min}}{x_{\max}-x_{\min}} \end{array}$$

where $x_{\min}$ is the minimum value and $x_{\max}$ is the maximum value. Standard scaling was used in this paper, so the amplitudes of the heartbeats were centered around the mean and distributed over the standard deviation of the data samples.

(3) $$x_{\mathrm{scaled}}=\frac{x-\mu }{\sigma }$$

where μ is the mean and σ is the standard deviation of the data.

#### 3.3.4. Data Augmentation/Oversampling

Since one heartbeat was extracted per image, we only had 928 samples in the data. This number was too small to train an accurate and generalized deep learning model. Moreover, there were fewer data samples for some classes than others, which could hamper model accuracy. The issue of class imbalance was addressed by employing SMOTE, which increases the samples of the minority classes using synthetic oversampling. This increased the number of samples in the minority class so that each class had an equal number of samples.

In the present work, we utilized SMOTE to increase all training data instead of only the minority class. Since the CPEIC cardiac dataset has one normal class and the other three classes belong to abnormal classes, we divided the data into binary classes. Making the data binary increased the samples in the abnormal class because it contained the data of all three abnormal classes. This made the normal class a minority, so SMOTE was applied to balance the dataset by synthetically oversampling the normal class examples. Then, separating the abnormal class into its individual classes turned the normal class into a majority class, and the individual abnormal classes became a minority. Applying SMOTE again to oversample the minority classes resulted in a larger dataset. This dataset was then used for training our deep-learning models. Since data augmentation was applied on the training set only, the test set did not have any synthetic data. The test set contained samples from the original dataset separate from the training set; therefore, the data augmentation process did not affect the model’s generalization.

In the following subsections, we evaluate popular machine learning models on the CPEIC cardiac dataset and then discuss the proposed deep learning models.

### 3.4. Machine Learning Methods

Numerous machine learning models were used for training on the CPEIC cardiac dataset, including the random forest classifier [48], k-nearest neighbors (kNN) classifier [49], and decision tree classifier [50]. To train all these models, Python’s library scikit−learn was used, which provides ready-to-use models that only require their hyper-parameters to be set.

The decision tree method is a famous machine learning technique for classification. It works by splitting the feature space based on some splitting criteria recursively until a stopping criterion is met. In this work, to train the decision tree classifier, the Gini index was used as the criteria for splitting. The accuracy and F1 score achieved on the CPEIC cardiac dataset using this method were 67.3% and 67.7%, respectively.

Random forest is an ensemble machine learning technique consisting of numerous decision trees. The individual predictions of the decision trees are aggregated or combined using an ensemble algorithm to obtain the final prediction. The individual decision trees are trained on a random subset of the data. For the random forest classifier, the hyper-parameters were set as follows: the number of trees was equal to 100, the maximum depth was also set to 100, and the splitting criteria used was the Gini index. The random forest classifier achieved an accuracy of 80.9% and an F1 score of 80.8% on the CPEIC cardiac dataset.

The k-nearest neighbor or kNN classifier works by assigning an input data point a class label based on the label of k data points in the neighborhood of the query point. The label is assigned based on the majority class. The choice of k depends on the dataset and can be optimized by validation. Three nearest neighbors were used in this method for classification on the CPEIC cardiac dataset. Using 2 neighbors or more than 3 resulted in poor accuracy. Using the three nearest neighbors, the accuracy and F1 score were 79.3% and 79.5%, respectively.

### 3.5. Proposed Deep Learning Methods

Various deep learning architectures were implemented and experimented on the CPEIC cardiac dataset. In signal processing, the convolution operation is used for feature extraction and the analysis of signals [51]. Since the ECG heartbeat signals are highly correlated in the time axis, applying convolution filters captures temporal correlations and extracts meaningful features for classification. Here, 1D convolutions are used in all of our proposed DL methods, as elaborated in the following subsections.

#### 3.5.1. Convolutional Neural Network (CNN)

**Figure 8:**
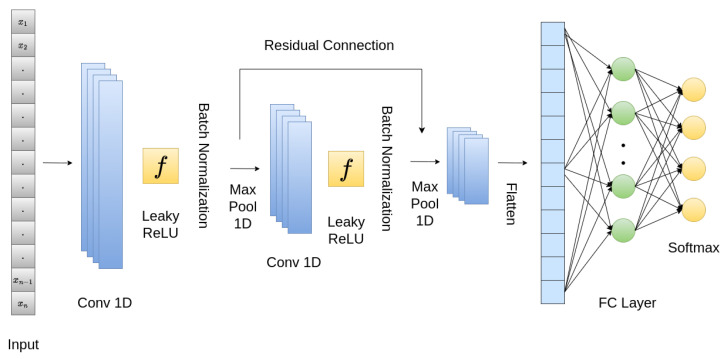
Proposed CNN architecture.

The proposed CNN contained a stack of 1D convolutional layers and fully connected (FC) layers at the end. The overall architecture is shown in Figure 8. The input to the model was a 234-dimensional vector that was passed through a 1D convolutional layer with Leaky ReLU as an activation function, whose slope coefficient α was 0.001. Leaky ReLU is expressed mathematically as:

(4) $$f\left(x\right)=\left\{\begin{array}{cc} x, & \mathrm{if}\;x\geq 0\\ \alpha x, & \mathrm{if}\;x<0 \end{array}\right.$$

To avoid overfitting and make the model more generalizable, batch normalization layers were used after the convolutional layers. To reduce the vector’s spatial dimension, one-dimensional max-pooling layers were used. A skip connection was applied as well, inspired by ResNet [52]. For this purpose, the features from the two convolutional layers were concatenated. Following the flattening of the output from the convolutional layers to obtain a one-dimensional feature vector, a fully connected layer was added, and subsequently, a softmax layer was added with four heads for the classification of cardiac diseases.

For training, *adam* [53] was used as an optimizer function with a learning rate of 0.0001. Adam merges the advantages of AdaGrad and RMSProp, enabling it to dynamically adapt the learning rate for individual parameters by considering the estimated first and second moments of the gradients. It is also computationally efficient and requires little memory. Sparse categorical cross-entropy loss was used to monitor the learning of our network. The proposed CNN was trained using a batch size of 16 for 100 epochs.

#### 3.5.2. Long Short-Term Memory (LSTM) Network

Long short-term memory (LSTM) networks [54] are usually used for sequential data or data that have a dependency in the time domain. For example, in natural language processing (NLP), LSTM networks are used for text generation [55], translation [56], etc. In time series data, such as stock prices and weather prediction, LSTMs can be used for analysis and forecasting [57,58]. Similarly, LSTMs are highly effective for the detection of anomalies in time series data [59]. ECG data are also time-series data, so an LSTM network was trained to predict cardiac diseases based on heartbeats.

**Figure 9:**
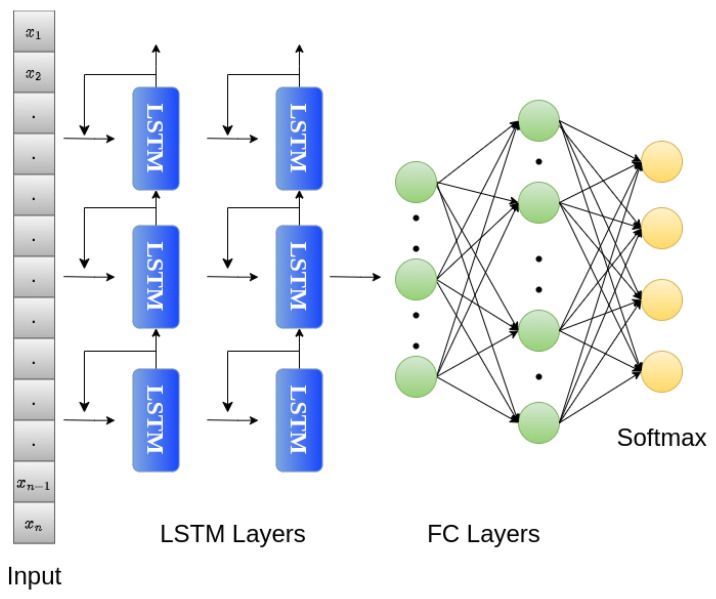
LSTM network.

In the proposed LSTM network, the 234-dimensional input was passed to two LSTM layers, with each layer containing 32 units. The activation function in each LSTM unit was tanh. After the LSTM layers, two fully connected or dense layers were added, with 256 and 512 units, respectively. Leaky ReLU with a slope coefficient of 0.001 was used as an activation function in the FC layers. The output layer at the end contained four nodes with a softmax activation function for classification. To train the network, the Adam optimizer was used with a learning rate of 0.001, and the loss function used was sparse categorical cross-entropy. The LSTM network was trained for 100 epochs with a batch size of 16. The proposed LSTM network is shown in Figure 9.

#### 3.5.3. Self-Supervised Learning Technique

**Figure 10:**
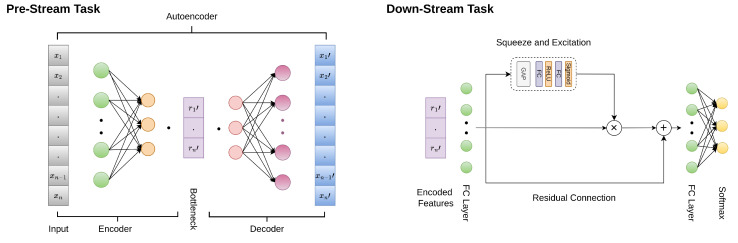
The self-supervised learning method.

Self-supervised learning techniques learn representations from data without requiring labels. These techniques are helpful when we have a large amount of unlabeled data in addition to few labeled data [60]. Since the CPEIC cardiac dataset is small compared to other benchmark datasets, such as the MIT-BIH dataset, using an SSL technique to extract features from the MIT-BIH dataset without labels can help meaningful features be learned, which can then be used in the classification model with labels on our smaller dataset. The self-supervised technique contained two tasks: a pre-stream task and a downstream task. The SSL model was trained on data without labels in the pre-stream task so that it learned representations from the data. Once the features were extracted from the pre-stream task, a classifier was trained with labels on the learned features. The overall flow of our SSL method is shown in Figure 10.

- **Pre-Stream Task**

For the pre-stream task, an autoencoder [61,62] was used. An autoencoder is a self-supervised technique that learns efficient representations by training a neural network for the reconstruction of the original input from a compressed representation. An encoder and a decoder are the two components of an autoencoder.

***Encoder***

The encoder takes the input and learns a compressed feature using a series of layers. The dimensions of the layers decrease at each step. The final layer of the encoder is called the bottleneck, which captures the important features of the data [63].

The proposed encoder consisted of two dense layers; the first layer had 468 neurons, and the second had 234 neurons. Subsequent to each layer, a batch normalization layer was incorporated. The activation function employed after each layer was Leaky ReLU, except for the bottleneck layer, where it was linear. The bottleneck consisted of half of the neurons present in the input dimension, i.e., 117, as our input size was 234.

***Decoder***

The learned representations from the bottleneck layer were used by the decoder to reconstruct the original input. The decoder also used a series of layers, but it was the mirror of the encoder layers in reverse. The final layer of the decoder needed to learn the representations that should match the input layer. Therefore, a reconstruction loss such as the mean squared error was used at the output layer [62,63].

The decoder of the proposed autoencoder was again made up of two levels with 234 neurons and 468 neurons, respectively. After each layer, a batch normalization layer was added, and Leaky ReLU was used as the activation function. The output layer had 234 neurons since we tried to reconstruct the input. Linear activation was used in the output layer.

***Training***

The autoencoder was trained on a combination of the MIT-BIH arrhythmia and CPEIC cardiac datasets without labels. Both datasets were concatenated to create an augmented dataset. These data were then divided into training and test sets. The model was trained for 10 epochs with the Adam optimizer. After training, we saved the trained encoder to extract the learned features for use in the next step.

- **Down-stream task or fine-tuning**

After training the autoencoder on the augmented dataset, a novel model was created for the classification of the encoded features. This model can be referred to as a ‘classifier’. The structure of the classifier is shown in Figure 10 in the down-stream task. It contained an FC layer with 128 neurons with ReLU activations. Then, attention layers were added, i.e., squeeze and excitation layers. The attention layer’s output and the previous dense layers were concatenated and passed through another dense layer with 128 neurons. After flattening, the final output layer with softmax activation was added with 4 neurons for classification with the CPEIC dataset.

***Training***

First, encoded features were extracted from the saved encoder from the pre-stream task. The encoded features were then passed as input to the classifier. The classifier was trained for 75 epochs with a batch size of 16. The Adam optimizer with a learning rate of 0.001 was used for training, and categorical cross-entropy loss was used to monitor the classifier’s learning.

## 4. Results and Discussion

The classification results of the machine learning and deep learning models are presented in this section. First, the performance metrics on which the models are evaluated are briefly discussed, and then the results obtained on the digitized CPEIC cardiac dataset are presented.

### 4.1. Performance Metrics

There are various quantitative measures to evaluate the performance of a classification model, including accuracy, precision, recall, specificity, and the F1 score. Accuracy is the most common standard for measuring the performance of ECG classification models. The F1 score is also commonly employed to evaluate classification models because it provides a balanced measure of precision and recall.

#### 4.1.1. Accuracy

Accuracy is a metric that is commonly used to evaluate classification models; it measures the correctly predicted or classified instances from the total samples. We can calculate accuracy in terms of true positives (TP), false positives (FP), true negatives (TN), and false negatives (FN) as:

(5) $$Accuracy=\frac{TP+TN}{TP+TN+FP+FN}$$

Alternatively,

(6) $$Accuracy=\frac{No.\;of\;Correct\;Predictions}{Total\;no.\;of\;Predictions}$$

#### 4.1.2. F1-Score

The F1 score, also referred to as f-score or f-measure, is another commonly used evaluation metric for classification tasks. The F1 score combines precision and recall and provides a balanced measure of a model’s accuracy using the harmonic mean of precision and recall. In terms of precision and recall, the F1 score is calculated as:

(7) $$F1\text{-}\mathrm{Score}=2*\frac{Precision\times Recall}{Precision+Recall}$$

where

(8) $$\begin{array}{c} Precision=\frac{TP}{TP+FP}\;,\;Recall=\frac{TP}{TP+FN} \end{array}$$

The recall is also known as sensitivity or the true-positive rate (TPR). The F1 score provides a good evaluation particularly when there is a class imbalance or when precision and recall both are important.

#### 4.1.3. Area under the Precision–Recall Curve (PR-AUC)

The PR-AUC is a valuable metric used to evaluate the performance of classification models, especially in scenarios where a class imbalance exists [64]. The PR-AUC shows the area under the curve of precision plotted against recall. It assesses a model’s ability to differentiate between classes by considering both precision and recall. A higher PR-AUC value indicates better model performance. The proposed CNN is evaluated against PR-AUC for both the multiclass and binary class scenarios.

### 4.2. Classification Results on the CPEIC Cardiac Dataset

#### 4.2.1. Machine Learning Models

This section shows the results of the machine learning models used for the classification of cardiac diseases using the digitized form of the CPEIC cardiac dataset. The accuracy of the decision tree classifier was 67.3%, and its F1 score was 67.7%. Among the machine learning models, this model achieved the lowest accuracy. Since the decision tree method is a single-tree structure, it was unable to capture the relationships in the ECG sequence. The random forest classifier obtained an accuracy of 80.9% and an F1 score of 80.4%. Its accuracy is better than the decision tree classifier’s because it used an ensemble of decision trees, thus capturing complex features of the data. The kNN classifier achieved an accuracy of 79.3% and an F1 score of 79.4%, which is better than the decision tree classifier. The possible reason for kNN performing better than the decision tree classifier is that the data contained distinctive features that helped the kNN methodology classify it easily and accurately. The results are tabulated in Table 6.

#### 4.2.2. Proposed Deep Learning Models

Two types of experiments were performed using the proposed CNN on the CPEIC cardiac dataset: binary classification and multiclass classification. In the binary classification case, two scenarios were experimented with. First, the multi-class classification task was converted into a binary classification task by assigning all abnormal classes a single label, A, referred to in this scenario as “N vs. A”. The proposed CNN model was trained on N vs. A and achieved ∼89% accuracy. The F1 score achieved in this task was 89.3%. In the second scenario, the CNN was trained on the N and MI classes only; we refer to this scenario as “N vs. MI”. The accuracy and the F1 score of the proposed CNN on N vs. MI were both 94.2% and the PR-AUC was ∼0.94.

In the multiclass classification of cardiac diseases with all four classes, state-of-the-art results were achieved using the proposed CNN. The achieved accuracy using the proposed CNN was 91.8%, and the F1 score was also 91.8%. The PR-AUC in this case was ∼0.91. The per-class accuracies of each of the four cardiac classes are also shown in Table 7.

The geometric mean (G-mean) was computed for the CNN in the N vs. Mi and multiclass scenarios to check the model’s generalization ability. It provides a balanced view of model performance by considering both sensitivity (recall) and specificity. The G-mean for the N vs. MI case was ∼0.93, and for the multiclass case, it was ∼0.90.

LSTMs are recurrent neural networks that capture long-term dependencies in sequential data. Hence, LSTM layers were used for our ECG classification tasks, but these failed to yield promising results. The probable reason for this outcome is that our data consisted of individual heartbeats, so the sequences are small and the temporal dependencies are relatively simple. LSTMs usually work better on longer sequential data by capturing long-term dependencies in the data. Consequently, the LSTM network did not prove to be the best model for cardiac disease classification using individual ECG heartbeats. The accuracy and F1 scores of the LSTM network on the CPEIC dataset were found to be around 80%.

The proposed SSL-based technique, which used an autoencoder on unlabeled data to extract learned features and then fine-tuning a classifier on labeled data, has shown poor results on the CPEIC cardiac dataset. After training the classifier for 75 epochs in the downstream task, it achieved an accuracy of 76.0% and an F1 score of 75.5%. The accuracy was low because the model was complex, and hence, it was overfitting on this dataset.

The results of the proposed deep learning techniques are summarized in Table 8.

Among the proposed deep learning models, the CNN’s performance on the digitized dataset is clearly better compared to the other models. The introduction of skip connections in the CNN allowed features from previous layers to be re-used, and batch normalization reduced overfitting, improving the model’s performance. Although the LSTM and SSL-based models are more complex, they could not perform well on the dataset. This shows that applying a complex model does not necessarily provide good results. The performance of the model is dependent on the data, as well as the problem.

#### 4.2.3. Inference Time Using the Proposed CNN

The inference time needed to predict cardiac diseases using the digitized ECG heartbeats was very brief. The time needed for the prediction of a single example using the proposed CNN model was observed. It accurately predicted the class in ∼0.1 s on Google Colab’s Intel Xeon CPU with 2 vCPUs and 13GB of RAM, which is perfectly suitable for the real-time monitoring of ECG data by directly deploying the model on an ECG machine.

## 5. Conclusions and Future Directions

In this work, an efficient and accurate model is developed for the detection of cardiac diseases using digitized ECG signals from images. First, ECG images of cardiac diseases are digitized using an open-source digitization tool to obtain a numerical time-series format. Different deep learning models are proposed and trained on the CPEIC cardiac dataset, including a CNN, an LSTM, and an SSL-based model using autoencoders. Among the proposed models, the CNN performs best, giving an accuracy of ∼91.8% and a fast inference time of one-tenth of a second, making our model suitable for the direct and real-time monitoring and classification of ECG signals on an ECG machine. Summarizing the main contributions of this research, the time-series format of the CPEIC cardiac dataset is employed, deep learning models are proposed for the efficient and real-time prediction of cardiac diseases, and the proposed CNN model can be deployed on an ECG machine for direct monitoring with high accuracy.

The dataset used in this study for the classification of different cardiac diseases is small. Creating a generalizable and accurate classification model requires a large amount of data, along with different configurations of the network. For future work, the dataset can be increased by collecting more data, as well as exploring data augmentation techniques for time series data. Additionally, one can explore using all the heartbeats from the image to obtain more data instead of digitizing only one heartbeat from lead II. In the self-supervised technique, a dense autoencoder is employed, where only dense layers are used in the network. To extend our current work, convolutional autoencoders using convolutional layers can be explored to compare the performance of the autoencoders. Moreover, experiments can be performed on the SSL-based model using different sizes of the bottleneck in the encoder part of the autoencoder.

## Figures and Tables

**Table 1:** Summary of machine learning approaches for the classification of ECG data.

Article	Year	Methodology	Dataset	Classes	Accuracy	F1 Score
[24]	2022	Optimized decision tree classifier with an adaptive boosting mechanism	MIT-BIH	6	98.77	93.85
[25]	2021	Ensemble of SVM and random forest	MIT-BIH	5	98.21	96.4
[28]	2022	Random forest on features from CNN	MIT-BIH	3	96	88.3
[29]	2020	Ensemble of kNN, DT, ANN, SVM, and LSTM	MIT-BIH	5	98.06	-
			PTB	2	97.66	96.99

**Table 2:** Summary of deep learning approaches for the classification of ECG data.

Article	Year	Methodology	Dataset	Classes	Accuracy	F1 Score
[3]	2022	CNNs with dropout and early stopping	MIT-BIH	10	95.7	-
[21]	2021	1D version of ResNet-50	PhysioNet 2021	23	52.0	-
[40]	2022	VGG-16 on images format of MIT-BIH	MIT-BIH	3	89	89
[41]	2021	SSD-MobileNet v2 used on 12-lead ECG images	CPEIC	4	98	96.1
[42]	2022	Modified version of LeNet-5	CPEIC	4	98.38	99
[43]	2022	MobileNet v2 and VGG-16	CPEIC	4	95	95
[36]	2023	LSTM network	MIT-BIH	5	99.64	98.18
[37]	2019	LSTM network	MIT-BIH	5	99	99
[20]	2020	Transformer neural network	PhysioNet 2020	27	53	-
[39]	2022	One-shot learning with domain adaptation for wearable devices	MIT-BIH	2	98.2	92.8
[14]	2022	SSL-based multi-modality method	PhysioNet 2020	25	48.9	62.1
[33]	2023	1D CNN	MIT-BIH	4	99	93
[34]	2023	Transfer learning and ensemble approach on 1D and 2D CNNs	MIT-BIH	5	94	92

**Table 3:** Beat annotations in the MIT-BIH arrhythmia database.

Label	Description
N	Normal beat
R	Right bundle branch block beat
L	Left bundle branch block beat
B	Bundle branch block beat (unspecified)
a	Aberrated atrial premature beat
A	Atrial premature beat
J	Nodal (junctional) premature beat
V	Premature ventricular contraction
S	Supraventricular premature or ectopic beat (atrial or nodal)
r	R-on-T premature ventricular contraction
e	Atrial escape beat
F	Fusion of ventricular and normal beats
j	Nodal (junctional) escape beat
E	Ventricular escape beat
n	Supraventricular escape beat (atrial or nodal)
/	Paced beat
Q	Unclassifiable beat
f	Fusion of paced and normal beats
?	Beat not classified during learning

**Table 4:** ECG image dataset of cardiac patients (CPEIC cardiac dataset).

Label	Description	No. of Images
N	Normal person	284
MI	Myocardial infarction patient	239
AHB	Patient with abnormal heartbeat	233
PMI	Patient with a previous history of MI	172

**Table 5:** Pros and cons of image-based vs. time series ECG classification.

Data Format	Pros	Cons
Images	Image-based techniques can be used Transfer learning of large-scale image classification models can be used Image-based data augmentation	Some fine-grained temporal information may be lost Large data size Computationally more complex
Time Series	Temporal information Direct and real-time monitoring Signal processing techniques can be applied Smaller data size	Limited use of image-based techniques Risk of high variance or overfitting

**Table 6:** Classification performance of machine learning models on four cardiac classes in the CPEIC cardiac dataset.

Model	Performance Metrics (%)	
	Accuracy	F1 Score
Decision tree	67.3	67.7
Random forest	**80.9** *	**80.4** *
kNN	79.3	79.4

* Best result in machine learning models.

**Table 7:** Per-class accuracy of cardiac diseases using CNN.

Class	Accuracy (%)
N	94.6
MI	90.7
PMI	88.9
AHB	90.7

**Table 8:** ECG classification results of the proposed deep learning methods on the CPEIC cardiac dataset.

Model	Classification Task	Classes	Accuracy (%)	F1-Score (%)	PR-AUC
CNN	Binary (N vs. A)	2	89.2	89.3	-
	Binary (N vs. MI)	2	**94.2** *	**94.2** *	∼0.94
	Multiclass	4	**91.8** ^ †^	**91.8** ^ †^	∼0.91
LSTM	Multiclass	4	78.6	78.4	-
SSL	Multiclass	4	76.0	75.5	-

* Best results in binary case. ^†^ Best results in multiclass case.

## Data Availability

The data may be requested by reaching out to authors through email.
